# Longitudinal Spatial Analysis of Health-Related Quality of Life in Mississippi: Clustering, Trends, and Socioeconomic Drivers

**DOI:** 10.5888/pcd23.260175

**Published:** 2026-09-10

**Authors:** Jae Eun Lee, JungHye Sung, Ji-Young Lee

**Affiliations:** 1 College of Health Sciences, Jackson State University, Jackson, Mississippi; 2 University of South Florida, Sarasota, Florida

## Abstract

**Introduction:**

Mississippi ranks among the lowest US states in health-related quality of life (HRQoL), with disparities most severe in rural and socioeconomically disadvantaged counties, particularly the Mississippi Delta. Understanding long-term spatial and temporal patterns is critical for equity-focused public health strategies. We aimed to describe geographic disparities in HRQoL across Mississippi’s 82 counties from 2015 through 2025, identify persistent hot spots of poor health, assess convergence with national averages, and inform tailored interventions.

**Methods:**

We used County Health Rankings & Roadmaps data (2015–2025) to construct an annual principal component analysis–derived composite HRQoL index from Behavioral Risk Factor Surveillance System measures (poor/fair health, physically unhealthy days, mentally unhealthy days). Local indicators of spatial association (LISA), emerging hot spot analysis (EHSA), and a spatial autoregressive lag model with year fixed effects assessed clustering, spatiotemporal trends, and predictors.

**Results:**

We found persistent disadvantage in poor/fair health in Mississippi (stable gap of 6 to 7 percentage points above national averages) but recent convergence in physically unhealthy days and reversal in mentally unhealthy days (fewer days in disadvantaged subgroups). LISA identified a persistent hot spot in the Delta and contraction of cold spots along the Gulf Coast by 2025. EHSA showed sporadic and consecutive hot spots concentrated in the Delta. The spatial autoregressive lag model confirmed significant dependence (ρ = 0.13, *P* < .001), with adult smoking and uninsurance as leading modifiable predictors.

**Conclusion:**

Mississippi’s HRQoL trajectory shows symptom-based improvements but persistent structural inequities and geographic disparities. Expanding smoke-free policies, strengthening primary care access, and increasing health insurance coverage are high-impact strategies to reduce disparities and advance health equity in the South.

**Summary**

**What is already known on this topic?**

Mississippi ranks poorly in health-related quality of life (HRQoL), with persistent disparities in rural and socioeconomically disadvantaged areas, such as the Delta, linked to smoking, uninsurance, and structural inequities.

**What is added by this report?**

This longitudinal analysis (2015–2025) used a principal component analysis–derived composite HRQoL index and advanced spatial methods to show symptom-based improvements (convergence in physically and mentally unhealthy days, reversal in disadvantaged subgroups) but persistent gaps in self-rated health and hot spots of poor health in the Delta.

**What are the implications for public health practice?**

Tailored interventions in the Delta could address modifiable drivers (smoking, uninsurance), convert recent gains into lasting improvements, and advance health equity in the South.

## Introduction

Health-related quality of life (HRQoL) is a multidimensional construct encompassing physical, mental, and social well-being and is commonly assessed by using the Centers for Disease Control and Prevention’s Healthy Days measures from the Behavioral Risk Factor Surveillance System (BRFSS) (1). HRQoL is strongly influenced by socioeconomic conditions — including income, education, rural residence, and access to health care — that shape chronic disease risk and contribute to persistent inequities across US communities (2,3).

Mississippi consistently ranks among the lowest US states in HRQoL and related health outcomes, with particularly severe disparities in rural and socioeconomically disadvantaged counties, especially those in the Mississippi Delta. Rates of poor physical and mental health days, poor or fair self-rated health, and chronic conditions such as diabetes and smoking-related diseases are higher in Mississippi than in the rest of the US (4,5). These gaps reflect long-standing structural inequities, limited health care access, and negative social determinants of health, with life expectancy in some rural areas lagging several years behind national norms (6).

Despite growing research on HRQoL, substantial gaps remain in longitudinal spatial analyses of multidimensional HRQoL, particularly in states with stark health disparities, such as Mississippi. National studies have identified clusters of low HRQoL in the southeastern US, including the Delta (3,7). However, applications of advanced spatial clustering methods — such as local indicators of spatial association (LISA) and emerging hot spot analysis (EHSA) — to BRFSS-derived County Health Rankings & Roadmaps (hereinafter, County Health Rankings) data are scarce. Most prior work has emphasized single-year or disease-specific outcomes rather than broad, temporal trends in composite HRQoL, leaving policymakers without detailed insights for equity-focused interventions amid postpandemic shifts in HRQoL indicators and health care access.

This study addresses these gaps by examining longitudinal trends and geographic disparities in HRQoL across Mississippi’s 82 counties from 2015 through 2025. Using a principal component analysis (PCA)–derived composite HRQoL index and advanced spatial analyses, we aimed to identify persistent hot spots of poor HRQoL, evaluate convergence of average scores in Mississippi with national averages, and uncover socioeconomic and behavioral drivers to inform precise, equity-focused public health strategies and chronic disease prevention in regions with a high burden of poor HRQoL.

## Methods

### Study setting and data source

We obtained county-level data for Mississippi from the County Health Rankings program for release years 2015 through 2025 (8). This program provides standardized annual measures derived from national surveys and administrative sources, including the BRFSS, National Center for Health Statistics mortality files, and US Census Bureau population estimates. We restricted analyses to Mississippi’s 82 counties and accessed data in December 2025. All data were publicly available and deidentified; therefore, institutional review board approval was not required.

### HRQoL measures and composite index

We used 3 core HRQoL measures from County Health Rankings: percentage of adults reporting fair or poor health, average number of physically unhealthy days in the past 30 days, and average number of mentally unhealthy days in the past 30 days (8). HRQoL measures are derived from BRFSS and use validated small-area estimation techniques for reliable county-level estimates (9,10). Higher values indicate poorer HRQoL. Because of multiyear BRFSS aggregation, each County Health Rankings release has a lag of 2 to 3 years (eg, the 2025 release primarily reflects conditions in ~2022) (9,10).

We applied PCA annually to the 3 core HRQoL measures (2015–2025) to construct a multidimensional composite index. PCA is parsimonious, data-driven, and widely used in public health index construction to reduce dimensionality while minimizing subjective weighting. We considered alternative approaches, such as equal-weight indices and factor analysis; however, equal weighting imposes arbitrary assumptions, and factor analysis is more suited to latent construct modeling, whereas PCA provides strong internal consistency and interpretability.

Annual PCA was used to preserve year-specific covariance structures among HRQoL indicators, allowing the composite index to reflect contemporaneous relationships among physical, mental, and self-rated health. This annual approach is consistent with longitudinal public health analyses that use County Health Rankings data when indicator relationships may shift over time (eg, postpandemic mental health dynamics) (11). The first principal component was retained (higher values = worse HRQoL), explaining 59.2% to 97.4% of the total variance (mean, 86.2%). We merged the resulting scores into the Mississippi county-year data set. The composite showed strong construct validity; pooled Pearson correlations (*r*) were 0.90 for poor or fair health, 0.77 for physically unhealthy days, and 0.46 for mentally unhealthy days (all *P* values < .001).

### Covariates

We selected covariates to align with the major domains of County Health Rankings — health behaviors, clinical care, and social and economic factors (8). After assessing multicollinearity, we retained 9 variables for inclusion in the spatial autoregression (SAR) lag model: the ratio of population to dentists, preventable hospital stays, adult smoking prevalence, diabetes prevalence, injury-related death rate per 100,000, percentage of non-Hispanic Black residents, percentage of adults aged 65 years or older, percentage of adults without health insurance, and median household income. Missing values were imputed by using the median within each release year, and variance inflation factors indicated acceptable levels of multicollinearity (all <5).

### Subgroup analyses

We conducted subgroup analyses to assess whether recent HRQoL convergence differed by socioeconomic context. We used median cutoffs to dichotomize the following socioeconomic indicators annually: uninsurance, unemployment, educational attainment, median household income, child poverty, racial composition, and age structure. We calculated median values separately for Mississippi counties and all non-Mississippi counties for each release year, ensuring that high strata and low strata reflected year-specific distributions in each population (8). For each subgroup, we calculated pooled mean (SD) for the 3 core HRQoL measures across the 2023–2025 County Health Rankings releases, reflecting conditions from approximately 2020 through 2022. We computed mean differences (Mississippi counties minus non-Mississippi counties), *P* values, and Cohen *d* effect sizes to quantify disparities, convergence, or reversals across socioeconomic strata (28). This stratification approach parallels methods used in prior county-level analyses of premature mortality and health disparities in Mississippi (11).

### Spatial analyses

Spatial analyses used 2023 US Census TIGER/Line county boundaries. We constructed row-standardized Queen contiguity weights from county centroids for consistency across years (12). We assessed global spatial autocorrelation annually with Moran *I* to evaluate clustering, dispersion, or randomness of HRQoL values (13). LISA analyses then identified significant (*P* < .05) spatial clusters of county-level composite HRQoL scores and classified counties as high–high (hot spots), low–low (cold spots), high–low, or low–high (12) (Box).

**Box. Classification of Clusters of Health-Related Quality of Life (HRQoL) Identified by Local Indicators of Spatial Association (LISA) and Emerging Hot Spot Analysis (EHSA). Higher values indicate poorer HRQoL.**

**LISA**

**High–High (hot spot):** County with worse HRQoL than average, surrounded by counties with worse HRQoL than average.

**Low–Low (cold spot):** County with better HRQoL than average, surrounded by counties with better HRQoL than average.

**High–Low:** County with worse HRQoL than average, surrounded by counties with better HRQoL than average.

**Low–High:** County with better HRQoL than average, surrounded by counties with worse HRQoL than average.

**Not significant:** No statistically significant clustering or spatial outlier pattern detected.

**EHSA**

**New hot spot: **A county that became a significant hot spot in the most recent study period and had not been identified previously as a significant hot spot.

**New cold spot: **A county that became a significant cold spot in the most recent study period and had not been identified previously as a significant cold spot.

**Consecutive hot spot: **A county that has been a significant hot spot for a series of recent consecutive periods but not throughout the entire study period.

**Consecutive cold spot: **A county that has been a significant cold spot for a series of recent consecutive time periods but not throughout the entire study period.

**Sporadic hot spot: **A county that was identified as a significant hot spot in multiple nonconsecutive time periods, indicating intermittent clustering of poor HRQoL.

**Sporadic cold spot: **A county that was identified as a significant cold spot in multiple nonconsecutive time periods, indicating intermittent clustering of favorable HRQoL.

**Diminishing hot spot: **A county that has remained a significant hot spot over time but whose intensity has decreased significantly, indicating a weakening concentration of poor HRQoL.

**Intensifying hot spot: **A county that has remained a significant hot spot over time and whose intensity has increased significantly, indicating a strengthening concentration of poor HRQoL.

**Oscillating hot spot: **A county that alternated between hot spot and cold spot status during the study period, indicating unstable or highly variable spatial patterns.

**No pattern: **A county for which no significant spatiotemporal clustering pattern was detected during the study period.

We selected LISA because of its location-specific clustering information, essential for detecting persistent Delta hot spots and distinguishing them from outliers. LISA statistics used 999 permutations; choropleth maps applied a consistent scale across years for comparability.

EHSA, implemented via the pyehsa Python library, assessed temporal evolution by using the space–time Getis-Ord Gi* statistic with k = 2 temporal neighborhoods and 499 Monte Carlo permutations (14). We used the Mann–Kendall test to evaluate Gi* *z* score trends, classifying counties as new, consecutive, sporadic hot spots, sporadic cold spots, or no pattern (15). We chose EHSA because of its ability to detect emerging, intensifying, diminishing, or persistent patterns not visible in LISA alone.

### Spatial regression modeling

We performed spatial regression modeling to determine key predictors of county-level HRQoL while accounting for geographic dependence and spillover effects. Strong spatial clustering of HRQoL — especially persistent disadvantage in the Mississippi Delta — necessitated a spatial approach to obtain unbiased estimates and to distinguish local covariate effects from influences transmitted through neighboring counties. A SAR lag model with year fixed effects (2015 as reference) was estimated via maximum likelihood, using the PCA-derived composite HRQoL score as the dependent variable. This specification was selected for its strong performance in prior Mississippi analyses (11) and its ability to capture substantive spatial spillovers. The model included 9 covariates plus a row-standardized Queen-contiguity spatial lag of the outcome. Year fixed effects controlled for temporal trends, and diagnostics confirmed no multicollinearity (variance inflation factor <5) and minimal residual spatial autocorrelation. We addressed temporal dynamics by including year fixed effects in the SAR model to control for time-specific trends, and these dynamics were explicitly modeled in the EHSA framework, which incorporated a temporal neighborhood structure and evaluated trends by using the Mann–Kendall test.

### Sensitivity analyses

To evaluate the robustness of the HRQoL composite index and spatial modeling results, we conducted 3 sensitivity analyses. First, to assess potential effects of mild nonlinearity in the unhealthy-days measures, we applied a log(1 + *x*) transformation to the physically unhealthy days and mentally unhealthy days variables before PCA and reconstructed the composite HRQoL index. Second, to address potential concerns about comparability of annual PCA scores across years, we conducted a pooled PCA using all county-year observations from 2015 through 2025 and re-estimated the SAR lag model using the pooled composite score. Third, we fitted a Bayesian hierarchical spatiotemporal model using the Besag–York–Mollié 2 (BYM2) specification with weakly informative priors and No-U-Turn Sampler (NUTS) estimation to evaluate the robustness of findings under an alternative framework that explicitly models structured and unstructured spatial random effects, fully propagates uncertainty, and applies partial pooling to stabilize small-area estimates (16). Results from these analyses were compared with those of the primary annual PCA-based SAR model to assess consistency of spatial patterns, predictor effects, and model inferences.

## Results

### Trends in HRQoL measures

Adults in Mississippi consistently reported more poor physical health days than adults nationally, with gaps ranging from 0 days (2023 release, reflecting conditions in ~2020) to 0.81 days (2020 release, reflecting conditions in ~2017) (Table 1). Disparities were largest in the 2020 and 2021 releases but narrowed substantially thereafter.

**Table 1:** Trends in Average Health-Related Quality of Life Measures in Mississippi and Non-Mississippi Counties, 2015–2025^a^

Year	No. of poor physical health days in past 30 days			No. of poor mental health days in past 30 days			In general, poor or fair health		
	Mississippi	Non-Mississippi	Gap^b^	Mississippi	Non-Mississippi	Gap^b^	Mississippi, %	Non-Mississippi, %	Gap,^b^ percentage point
2015	4.37	3.81	0.56	4.15	3.54	0.61	23.2	17.1	6.1
2016	4.26	3.80	0.46	4.09	3.66	0.43	22.7	16.8	5.9
2017	4.57	3.90	0.67	4.32	3.77	0.55	24.0	16.8	7.2
2018	4.32	3.91	0.41	4.26	3.92	0.34	23.5	17.3	6.2
2019	4.32	3.91	0.41	4.26	3.92	0.34	23.5	17.3	6.2
2020	4.78	3.97	0.81	4.72	4.15	0.57	25.2	17.7	7.4
2021	5.01	4.37	0.64	5.25	4.66	0.59	26.6	19.9	6.6
2022	4.70	4.33	0.37	5.50	4.87	0.63	26.7	20.5	6.2
2023	3.52	3.52	0.00	4.56	4.81	−0.25	21.9	15.9	6.0
2024	4.15	3.89	0.26	5.04	5.22	−0.18	24.5	17.5	7.0
2025	4.48	4.46	0.02	5.37	5.65	−0.28	25.4	19.4	6.0

a Measures were derived from Behavioral Risk Factor Surveillance System and typically reflect conditions with a 2- or 3-year lag; for example, the 2025 release primarily uses 2022 data, while earlier releases aggregate multiple prior years.

b Mississippi counties minus non-Mississippi counties. Any inconsistency in math is due to rounding.

For poor mental health days, values in Mississippi increased across earlier releases, consistent with national trends. However, the increases in Mississippi counties were generally smaller than increases in non-Mississippi counties. As a result, the Mississippi–US gap narrowed over time and eventually reversed beginning with the 2023 release (reflecting conditions in ~2020–2022), with negative gaps of –0.18 (in 2024) to –0.28 days (in 2025). This pattern indicates a relative improvement compared with national trends rather than an absolute decline in the number of poor mental health days in Mississippi. In contrast, the percentage of adults in Mississippi reporting poor or fair health (range, 21.9%–26.7%) remained persistently higher than the percentage nationally (range, 15.9%–20.5%) across all releases, with a stable gap of approximately 6 to 7 percentage points, reflecting a chronic disparity in overall self-rated health.

### Subgroup differences in recent convergence of HRQoL measures

Of the 3 core HRQoL measures, poor mental health days showed the clearest reversal: in nearly every high-burden subgroup, Mississippi reported fewer mentally unhealthy days than comparable non-Mississippi counties (moderate to large effects, *d* = 0.23–0.83) (Table 2). The strongest reversals occurred in the most disadvantaged communities — those with a high rate of child poverty, low educational attainment, low income, unemployment, or a high percentage of non-Hispanic Black residents — indicating comparatively better recent mental well-being conditional on socioeconomic disadvantage.

**Table 2:** Subgroup Differences in Health-Related Quality-of-Life Measures Between Mississippi and Non-Mississippi Counties, 2023–2025^a^

Subgroup	No. of poor physical health days, mean (SD)				No. of poor mental health days, mean (SD)			
	Non-Mississippi	Mississippi	Gap^b^	Cohen *d*^ c^	Non-Mississippi	Mississippi	Gap^b^	Cohen *d*^ c^
**Uninsured children (% of children aged <19 y without health insurance)**								
Low	3.88 (0.80)	4.04 (0.59)	0.16 (0.05)^d^	0.20	5.25 (0.73)	4.95 (0.45)	−0.30 (0.04)^e^	0.41
High	4.03 (0.72)	4.06 (0.51)	0.03 (0.05)	0.04	5.20 (0.69)	5.03 (0.37)	−0.18 (0.04)^e^	0.26
**Uninsured adults (% of adults aged <65 y without health insurance)**								
Low	3.76 (0.77)	3.87 (0.57)	0.11 (0.05)^f^	0.14	5.13 (0.73)	4.90 (0.43)	−0.23 (0.04)^e^	0.32
High	4.15 (0.70)	4.23 (0.46)	0.08 (0.04)	0.11	5.32 (0.67)	5.08 (0.38)	−0.24 (0.04)^e^	0.36
**Unemployment (% of those aged ≥16 y who are unemployed but seeking work)**								
Low	3.72 (0.68)	3.85 (0.53)	0.13 (0.05)^d^	0.19	5.01 (0.68)	4.93 (0.40)	−0.08 (0.04)^f^	0.13
High	4.20 (0.77)	4.25 (0.50)	0.05 (0.05)	0.07	5.44 (0.68)	5.05 (0.42)	−0.39 (0.04)^e^	0.59
**Some college (% of adults aged 25–44 y with some postsecondary education)**								
Low	4.37 (0.68)	4.26 (0.48)	−0.11 (0.04)^f^	0.17	5.53 (0.65)	5.05 (0.41)	−0.48 (0.04)^e^	0.75
High	3.54 (0.60)	3.84 (0.53)	0.30 (0.05)^e^	0.51	4.92 (0.64)	4.92 (0.41)	0.00 (0.04)	0.01
**Median household income**								
Low	4.35 (0.71)	4.28 (0.48)	−0.07 (0.04)	0.10	5.51 (0.68)	5.04 (0.42)	−0.47 (0.04)^e^	0.70
High	3.56 (0.59)	3.82 (0.52)	0.25 (0.05)^e^	0.43	4.94 (0.62)	4.94 (0.41)	−0.00 (0.04)	0.01
**High school graduation (% of a ninth-grade cohort that graduates from high school in 4 years)**								
Low	3.91 (0.75)	4.16 (0.50)	0.25 (0.04)^e^	0.34	5.15 (0.72)	5.02 (0.40)	−0.13 (0.04)^e^	0.18
High	4.03 (0.79)	3.92 (0.57)	−0.11 (0.06)^f^	0.15	5.35 (0.68)	4.95 (0.44)	−0.41 (0.04)^e^	0.60
**Children in poverty (% of children aged <18 y living in poverty)**								
Low	3.53 (0.58)	3.85 (0.53)	0.31 (0.05)^e^	0.53	4.90 (0.62)	4.95 (0.42)	0.05 (0.04)	0.08
High	4.38 (0.68)	4.26 (0.49)	−0.12 (0.05)^d^	0.18	5.56 (0.64)	5.03 (0.41)	−0.53 (0.04)^e^	0.83
**% Non-Hispanic White (% of the resident population identifying as non-Hispanic White)**								
Low	4.04 (0.73)	4.19 (0.55)	0.15 (0.05)^d^	0.20	5.28 (0.65)	4.98 (0.43)	−0.30 (0.04)^e^	0.47
High	3.87 (0.78)	3.91 (0.52)	0.04 (0.05)	0.05	5.18 (0.77)	5.00 (0.40)	−0.17 (0.04)^e^	0.23
**% Non-Hispanic Black (% of resident population identifying as non-Hispanic Black or African American)**								
Low	3.86 (0.79)	3.89 (0.51)	0.03 (0.05)	0.04	5.11 (0.75)	4.99 (0.40)	−0.12 (0.04)^d^	0.16
High	4.05 (0.73)	4.21 (0.54)	0.16 (0.05)^d^	0.22	5.34 (0.65)	4.99 (0.43)	−0.36 (0.04)^e^	0.55
**% Below 18 years of age (% of the total resident population that is under 18 years of age)**								
Low	3.93 (0.74)	4.04 (0.50)	0.11 (0.05)^f^	0.14	5.27 (0.69)	4.96 (0.40)	−0.32 (0.04)^e^	0.46
High	3.98 (0.78)	4.06 (0.59)	0.08 (0.05)	0.10	5.18 (0.73)	5.02 (0.43)	−0.16 (0.04)^e^	0.22
**% 65 and older (% of the total resident population that is 65 years of age or older)**								
Low	3.99 (0.78)	3.98 (0.60)	−0.01 (0.05)	0.01	5.25 (0.68)	4.95 (0.42)	−0.30 (0.04)^e^	0.44
High	3.92 (0.75)	4.12 (0.49)	0.20 (0.05)^e^	0.27	5.21 (0.74)	5.02 (0.40)	−0.18 (0.04)^e^	0.25

a Data are pooled mean (SD) from County Health Rankings & Roadmaps releases 2023–2025 (reflecting approximately 2020–2022 conditions).

b Mississippi counties minus non-Mississippi counties.

c Cohen *d* values >0.5 indicate moderate to large effects.

d *P* < .01; determined by *t* test for mean differences.

e *P* < .001; determined by *t* test for mean differences.

f *P* < .05; determined by *t* test for mean differences.

Poor physical health days showed mixed convergence. The statewide gap narrowed, and many high-burden subgroups showed small or nonsignificant differences (eg, high rates of uninsurance and unemployment, high percentage of non-Hispanic White residents). Several disadvantaged strata even favored Mississippi (negative gaps), particularly groups with high rates of child poverty, low educational attainment, and low income. However, larger positive gaps persisted in advantaged subgroups (eg, low rate of child poverty, *d* = 0.53; high rates of some college education, *d* = 0.51), suggesting that convergence was driven primarily by improvements in Mississippi’s most socioeconomically disadvantaged counties. The measure for poor or fair health remained persistently worse in Mississippi than in non-Mississippi US counties across all releases.

### Spatial clustering patterns

**Figure 1:**
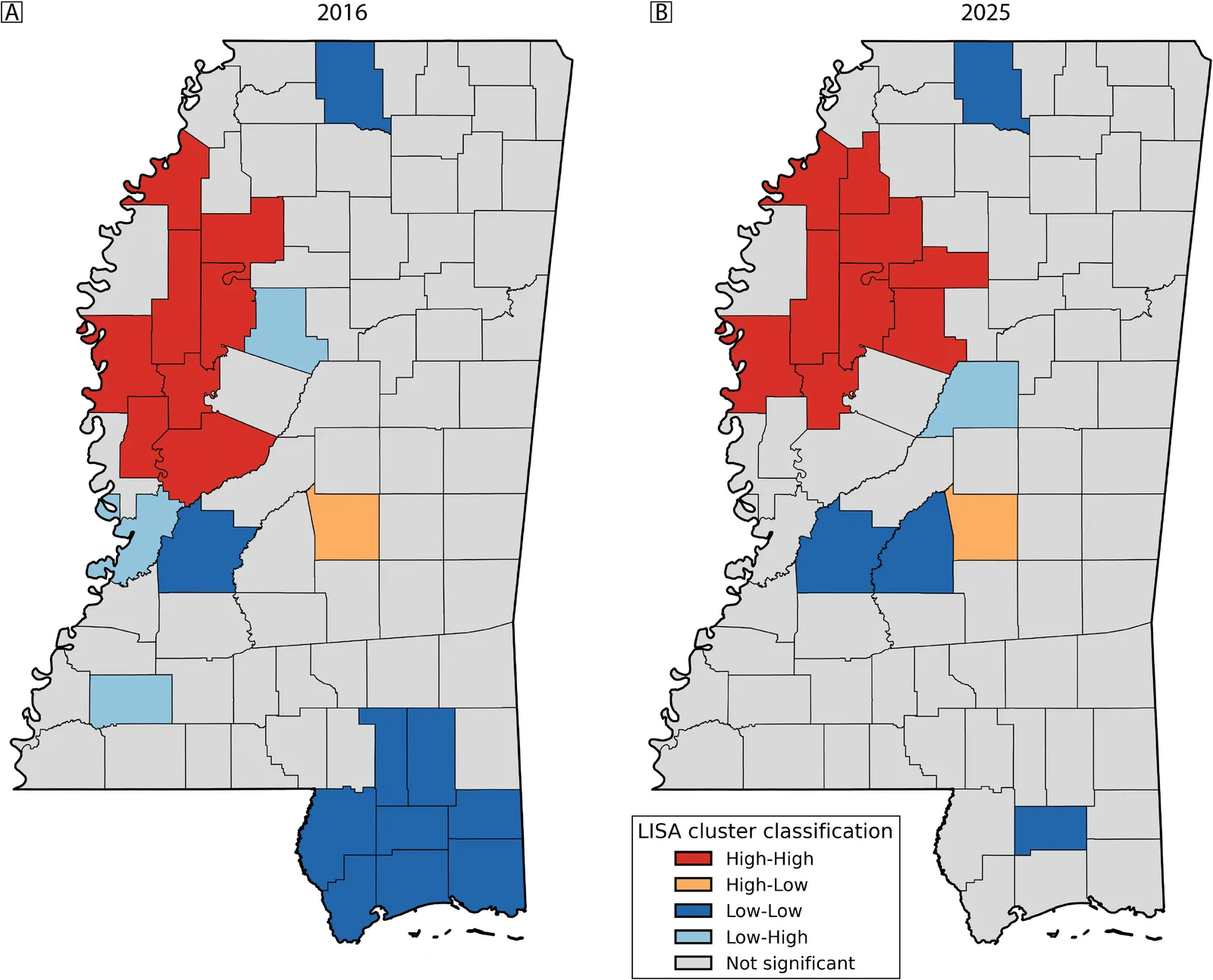
Local indicators of spatial association (LISA) cluster maps for the principal component analysis–based composite health-related quality-of-life score in Mississippi Counties. A) 2016; B) 2025. Data source: County Health Rankings & Roadmaps, 2015–2025 releases.

**Figure 2:**
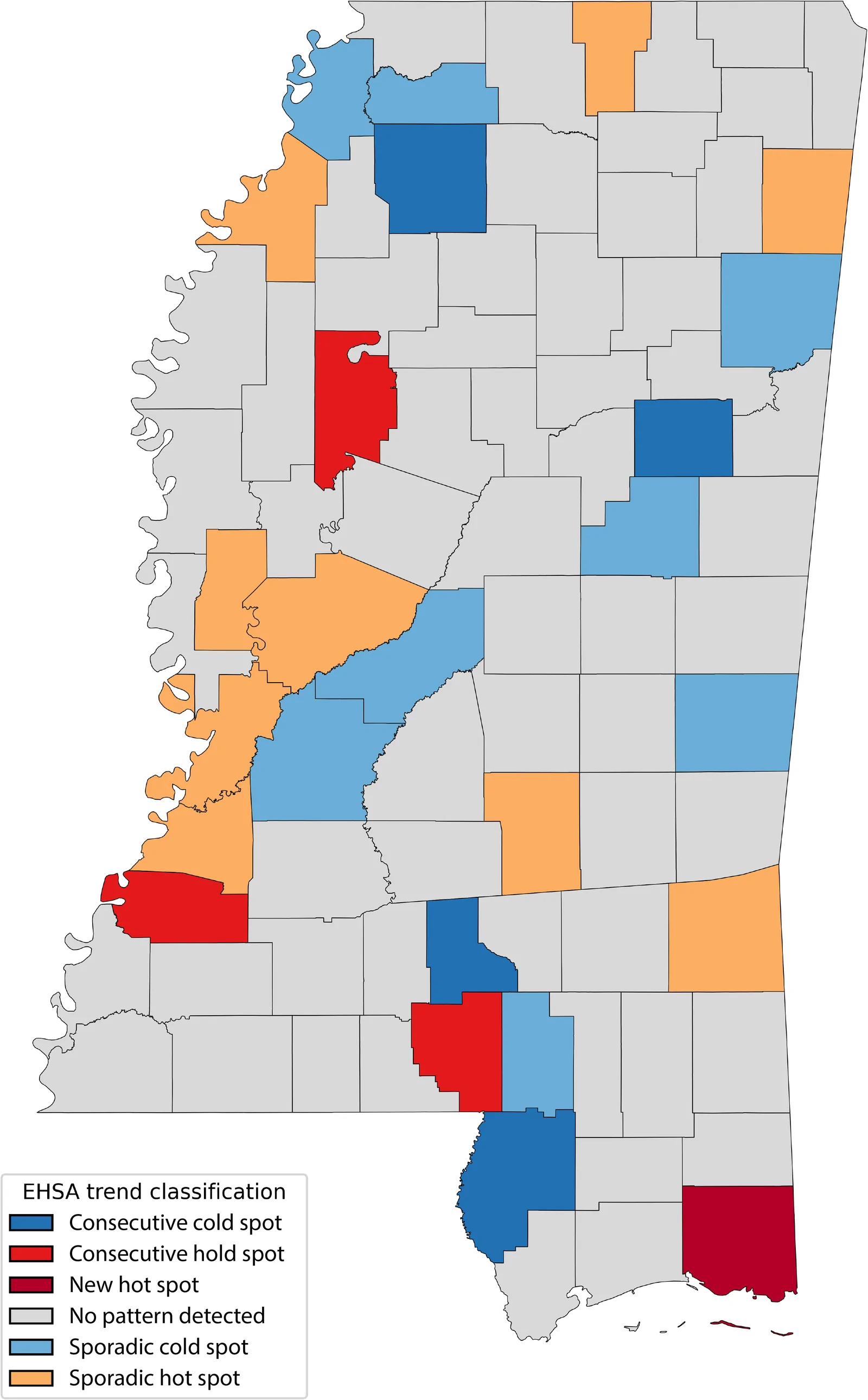
Emerging hot spot analysis (EHSA) of trends in health-related quality-of-life scores in Mississippi counties from 2015 through 2025. Data source: County Health Rankings & Roadmaps, 2015–2025 releases.

LISA and EHSA demonstrated persistent yet evolving geographic disparities in HRQoL in Mississippi (Figure 1 and Figure 2). LISA identified significant local clusters in 2016 and 2025. In 2016, we observed significant clusters in 22 counties, including a prominent hot spot (poor HRQoL) in 8 Delta counties and a cold spot (better HRQoL) in 10 counties along the Gulf Coast and central/suburban counties. By 2025, the number of significant clusters declined to 15 counties, with the Delta hot spot persisting and expanding to 9 counties, while the cold spot contracted sharply to 4 counties.

**Table:** 

LISA cluster classification	Counties	
	2016 (n = 22)	2025 (n = 15)
High-High	Coahoma, Humphreys, Leflore, Sharkey, Sunflower, Tallahatchie, Washington, Yazoo	Carroll, Coahoma, Grenada, Humphreys, Leflore, Quitman, Sunflower, Tallahatchie, Washington
Low-Low	Forrest, George, Hancock, Harrison, Hinds, Jackson, Marshall, Pearl River, Perry, Stone	Hinds, Marshall, Rankin, Stone
Low-High	Carroll, Franklin, Warren	Attala
High-Low	Scott	Scott

**Table:** 

EHSA trend classification	No. of counties	County names
Consecutive cold spot	4	Jefferson Davis, Oktibbeha, Panola, Pearl River
Consecutive hot spot	3	Jefferson, Leflore, Marion
New hot spot	1	Jackson
Sporadic cold spot	8	Hinds, Lamar, Lauderdale, Madison, Monroe, Tate, Tunica, Winston
Sporadic hot spot	9	Benton, Claiborne, Coahoma, Itawamba, Sharkey, Smith, Warren, Wayne, Yazoo
No pattern detected	57	All others

EHSA of trends (2015–2025) produced Gi* z-scores from –5.155 to 4.966, with 231 of 899 county-year observations significant at *P* less than or equal to .05 (25.7%). Most counties (69.5%) showed no detectable pattern, while detectable trends concentrated in the Delta: sporadic hot spots (9 counties), sporadic cold spots (8 counties), consecutive hot spots (3 counties), consecutive cold spots (4 counties), and 1 new hot spot in a southern coastal-adjacent county. We found no diminishing, intensifying, or oscillating hot spots.

### Quantitative confirmation of spatial patterns

The SAR lag model (Table 3) indicated substantial spatial dependence, with a positive and significant spatial lag coefficient (ρ = 0.125, *P* < .001), meaning that county HRQoL levels were influenced by those of neighboring counties even after adjusting for covariates. Key predictors of worse HRQoL included adult smoking (strongest effect), uninsurance, diabetes prevalence, percentage of non-Hispanic Black residents, injury-related deaths, and preventable hospital stays, while higher median household income was strongly protective. Neither the percentage of adults aged 65 years or older nor the population-to-dentist ratio showed significant associations.

**Table 3:** Spatial Autoregressive Model Results for the Principal Component Analysis–Based Health-Related Quality-of-Life Composite Score, Mississippi, 2015–2025^a^^,^^b^

Variable	Coefficient^c^ (SE)	*z* value	*P* value
Constant	−8.10 (0.58)	−14.05	<.001
Ratio of population to dentists	0	1.66	.10
Preventable hospital stays	0	2.58	.01
Prevalence of adult smoking, %	29.06 (1.13)	25.77	<.001
Prevalence of diabetes, %	3.12 (1.10)	2.84	.005
Injury-related deaths (per 100,000)	0	2.56	.01
% Non-Hispanic Black	0.99 (0.19)	5.25	<.001
% Aged ≥65 y	−1.26 (1.03)	−1.23	.22
Adults without health insurance, %	7.68 (1.19)	6.44	<.001
Median household income	0	−9.07	<.001
**Year^d^**			
2016	0.66 (0.11)	6.29	<.001
2017	0.92 (0.11)	8.52	<.001
2018	1.20 (0.12)	9.90	<.001
2019	1.00 (0.19)	5.39	<.001
2020	1.12 (0.18)	6.08	<.001
2021	0.08 (0.18)	0.41	.68
2022	0.78 (0.17)	4.69	<.001
2023	1.21 (0.15)	7.87	<.001
2024	1.27 (0.16)	8.08	<.001
2025	1.89 (0.17)	11.28	<.001
ρ (spatial lag)	0.13 (0.04)	3.38	<.001

a All covariates were obtained from County Health Rankings & Roadmaps releases (2015–2025), derived from the Behavioral Risk Factor Surveillance System, Centers for Medicare & Medicaid Services, Area Health Resources Files, National Center for Health Statistics, and American Community Survey, as documented by County Health Rankings & Roadmaps.

b N = 899 county-year observations. Pseudo *R* ^2^ = 0.840. Spatial pseudo *R* ^2^ = 0.840.

c Coefficients represent direct effects; higher values indicate worse health-related quality of life.

d 2015 is reference category.

Year fixed effects were generally positive and significant relative to 2015, with the largest increase in 2025 (coefficient = 1.893, *P* < .001), suggesting a worsening of composite HRQoL in the most recent period. The high pseudo *R* ^2^ (0.840) and the significant spatial spillover term underscore the model’s strong explanatory power for geographic variation in HRQoL.

### Sensitivity analyses

In the sensitivity analysis that assessed robustness to mild nonlinearity, the transformed composite correlated highly with the original (pooled *r* = 0.9992; year-specific *r* ≥ 0.9984) and produced nearly identical spatial patterns. Because results were unchanged, the primary analyses used the untransformed composite for interpretability and consistency with standard HRQoL reporting.

In the sensitivity analysis that comprised a pooled PCA using all county-year observations, results were highly consistent with the primary annual PCA specification. Spatial dependence remained significant (ρ = 0.121, *P* = .002), and key predictors — including adult smoking (β = 22.86, *P* < .001), uninsured adults (β = 6.13, *P* < .001), and percentage of non-Hispanic Black residents (β = 1.12, *P* < .001) — were unchanged in direction and significance. Model fit remained strong (pseudo *R* ^2^ = 0.878). These findings indicate that variation in PCA loadings across years did not materially affect substantive conclusions (Appendix).

The Bayesian hierarchical model produced consistent findings, confirming significant spatiotemporal patterning (spatial mixing ρₛ = 0.526; 95% high-density interval [HDI], 0.068–0.999). Rate of adult smoking (posterior mean β coefficient = 0.580; 95% HDI, 0.518–0.645), percentage of non-Hispanic Black residents (0.604; 95% HDI, 0.462–0.749), injury-related deaths (0.183; 95% HDI, 0.103–0.260), and dentist shortages (posterior mean β coefficient = 0.111; 95% HDI, 0.015–0.207) emerged as credible predictors of worse HRQoL, while uninsurance showed no credible effect (posterior mean β coefficient = –0.009; 95% HDI, –0.093 to 0.075).

## Discussion

This study provides a comprehensive longitudinal assessment of HRQoL in Mississippi from 2015 through 2025 using a PCA-derived composite index from County Health Rankings data. The trajectory was mixed: self-rated poor or fair health remained persistently higher in Mississippi than in the rest of the nation (stable gap of 6 to 7 percentage points), reflecting deep-rooted structural inequities documented in prior work (17–19). This persistent gap aligns with the dominant contribution of self-rated health to the first principal component, whereas convergence in symptom-based domains (physically and mentally unhealthy days) drove more favorable recent patterns, along with persistent geographic clustering of disadvantage identified in spatial analyses. In the most recent releases (2023–2025, reflecting conditions in ~2020–2022), gaps in physically unhealthy days narrowed and gaps in mentally unhealthy days reversed, with the latter favoring the state’s most socioeconomically disadvantaged subgroups (eg, high rate of child poverty, low educational attainment, and unemployment). These improvements should be interpreted cautiously because they reflect conditions only through ~2022 and precede full postpandemic recovery. The reversal may reflect community resilience and the buffering effects of social networks documented in disadvantaged populations (20,21), although causal inference is limited. However, the absence of parallel improvement in self-rated health suggests that short-term symptom relief has not translated into broader perceptions of well-being, likely due to continued exposure to poverty, racial inequities, and chronic underinvestment in health infrastructure. The divergence between persistently elevated self-rated health measures and more variable unhealthy-day metrics likely reflects differences in construct sensitivity and temporal responsiveness. Self-rated health captures broader, more stable perceptions of well-being shaped by long-standing structural conditions such as poverty, chronic disease burden, and access to care, whereas unhealthy-day measures are more sensitive to short-term fluctuations, including pandemic-related stressors, health care disruptions, and temporary socioeconomic supports. This distinction helps explain why recent improvements in symptom-based indicators did not translate into corresponding changes in overall health perception, which may require sustained structural change over longer periods of time. These findings translate into clear public health implications: counties identified as having a persistently high burden of poor HRQoL — particularly in the Mississippi Delta — are priority areas for place-based intervention strategies. The combination of sustained geographic clustering and strong socioeconomic predictors indicates that improvements in HRQoL are unlikely to emerge from individual-level interventions alone but instead require coordinated regional approaches addressing health care access, economic disadvantage, and behavioral risk factors. Although dichotomizing subgroups reduces precision, the presence of multiple significant contrasts with moderate to large effect sizes (Cohen *d* up to 0.83) indicates that the findings are robust and meaningful.

LISA further confirmed enduring geographic disparities. The Mississippi Delta consistently emerged as a stable hot spot of poor HRQoL in 2016 and 2025, underscoring entrenched structural disadvantage in this region. In contrast, the cold spot of better HRQoL along the Gulf Coast and suburban counties weakened substantially by 2025, shrinking from 10 to 4 counties. This erosion of relative advantages suggests either diffusion of modest statewide improvements in symptom-based HRQoL or persistent barriers that limited further gains in formerly advantaged regions, even as core Delta hot spots remained remarkably stable. From a policy perspective, this reinforces the need for geographically targeted interventions rather than uniform statewide strategies.

The SAR lag model quantified significant spillover effects and identified adult smoking and uninsurance as the strongest modifiable predictors of worse HRQoL, followed by diabetes prevalence and racial composition. Many Mississippi counties still lack comprehensive smoke-free ordinances, particularly in regions such as the Delta. Previous studies indicate that areas without such policies tend to have higher smoking prevalence and poorer health outcomes, which may contribute to persistently worse HRQoL across multiple indicators (22–24). Sensitivity analyses reinforced these findings: a log(1 + x) transformation of unhealthy-days measures produced a nearly identical composite (pooled *r* = 0.9992), and Bayesian hierarchical models confirmed significant spatiotemporal patterning while yielding more conservative covariate estimates (eg, uninsurance not credibly different from zero), likely due to spatial confounding absorbed by random effects (25,26). Although annual PCA introduces variation in component interpretation across years, sensitivity analyses using a pooled PCA — with a fixed component structure — produced substantively identical findings. Spatial dependence remained significant, and the same key predictors of HRQoL, including smoking, uninsurance, and socioeconomic composition, were consistently identified. These results strengthen confidence that the observed spatial and socioeconomic patterns reflect underlying population health processes rather than artifacts of index specification.

EHSA revealed additional intra-Delta complexity. Persistent hot spots dominated the region, reflecting long-standing structural disadvantage, yet sporadic hot spots (9 counties) and sporadic cold spots (8 counties) coexisted in close proximity. This heterogeneity reflects a high baseline burden of poor HRQoL driven by extreme poverty, limited health care access, chronic disease, and historical disinvestment, combined with county-level fluctuations from temporary economic shifts, differential public health interventions, hospital openings/closures, seasonal agricultural cycles, or BRFSS estimation variability. These oscillating patterns highlight the dynamic nature of inequities even in a severely disadvantaged region and demonstrate the value of longitudinal spatiotemporal tools like EHSA for detecting subtle shifts obscured in cross-sectional analyses. From a policy perspective, the presence of sporadic cold spots suggests that targeted, place-based interventions — such as expanded primary care access, tobacco control, and insurance enrollment — can produce measurable improvements even amid broader structural challenges. Sustained investment in high-variance counties could convert intermittent gains into persistent improvements, accelerating convergence with national trends and advancing health equity in the South.

Policy implications are direct and actionable. Expanding comprehensive smoke-free air policies statewide — particularly in the Delta, where protections are limited — represents a potentially high-impact strategy to reduce smoking-related HRQoL, as adult smoking was the strongest and most consistent predictor across both models (24). Local ordinances currently cover only about 37% of the population, and a statewide law is pending at the time of manuscript preparation. Similarly, Medicaid expansion — which Mississippi had not implemented during the study period (with 2025 bills failing in committee) — would address persistently high uninsurance rates and improve access to preventive and chronic disease care in Delta counties, even though Bayesian estimates were more conservative regarding uninsurance as a direct driver (27). Place-based investments targeting the persistent hot spots — tobacco control, diabetes prevention, and primary care enhancement — along with sustained support for social determinants of health are essential to translate recent symptom-based gains into durable improvements in overall well-being, particularly as relative advantages in other regions continue to erode.

### Strengths and limitations

Strengths of this study include its longitudinal design spanning 11 years of County Health Rankings & Roadmaps data, the construction of a robust PCA-derived composite HRQoL index (with sensitivity analyses confirming robustness to mild nonlinearity in recent years), the application of advanced spatial analytic techniques (including global Moran *I*, LISA clustering, and SAR modeling) (13) to elucidate persistent geographic patterns, and subgroup analyses that — despite some information loss from dichotomization — identified multiple significant subgroups with moderate-to-large effect sizes (Cohen *d* up to 0.83), demonstrating inferential robustness (28).

This study has several limitations, many inherent to the County Health Rankings & Roadmaps ecosystem and BRFSS-derived measures: a 2- to 3-year data lag potentially delays detection of recent HRQoL shifts; reliance on model-based small-area estimates for key covariates (eg, adult smoking and uninsurance rates) with varying precision may attenuate associations during rapid societal changes (29); the use of county-level data may obscure substantial within-county variability, particularly in larger or socioeconomically diverse counties, potentially limiting the precision of spatial inference (30); and self-reported measures may be susceptible to recall, social desirability, and cultural reporting biases (31).

Additional study-specific limitations include the PCA-derived composite index assuming linear relationships and being dominated by self-rated poor or fair health, which may underrepresent evolving multidimensional dynamics (eg, postpandemic mental health shifts) (32); dichotomization in subgroup analyses causing information loss and reliance on arbitrary cutpoints (33); the SAR lag model imposing homogeneous spatial dependence without allowing spatially varying coefficients or multilevel random effects, and not explicitly modeling temporal autocorrelation beyond year fixed effects, which may have led to underestimation of temporal dependence and limited the ability to capture dynamic changes over time (25,26); a predominantly cross-sectional panel structure limiting modeling of true temporal trajectories or lagged effects (34); and the ecological design precluding individual-level causal inference (35).

### Conclusion

Mississippi’s HRQoL trajectory reflects a tension between modest symptom-based improvements (observed through conditions in ~2020–2022) and enduring disparities in overall well-being. The persistent Delta hot spot and erosion of Gulf Coast advantages highlight the urgent need for targeted, place-based interventions to address structural inequities. Policy efforts should prioritize Delta-focused strategies, including expanded tobacco control (eg, comprehensive smoke-free policies), diabetes prevention, and expansion of health insurance coverage to tackle the key modifiable drivers identified in the SAR model. Sustained federal and state investments — building on pandemic-era supports — could accelerate convergence with national trends, but long-term progress will require confronting deep-rooted inequities to improve not just acute symptoms but holistic perceptions of health in the state’s communities most vulnerable to poor health outcomes.

## Appendix Sensitivity Analysis: Spatial Autoregressive Model Results for the Pooled Principal Component Analysis–Based Health-Related Quality-of-Life Composite Score, 2015–2025^a,b^

**Table:**

Variable	Coefficient^c^ (SE)	*z* value	*P* value
Constant	−7.22 (0.47)	−15.28	<.001
Ratio of population to dentists	0	1.79	.07
Preventable hospital stays	0	2.12	.03
Prevalence of adult smoking, %	22.86 (0.92)	24.76	<.001
Prevalence of diabetes, %	1.40 (0.90)	1.56	.12
Injury-related deaths (per 100,000)	0	2.34	.02
% Non-Hispanic Black	1.12 (0.16)	7.13	<.001
% Aged ≥65 y	−0.53 (0.84)	−0.63	.53
Adults without health insurance, %	6.13 (0.98)	6.27	<.001
Median household income^d^	0	−7.26	<.001
Year 2016	0.21 (0.09)	2.43	.02
Year 2017	1.08 (0.09)	12.25	<.001
Year 2018	0.95 (0.10)	9.62	<.001
Year 2019	0.83 (0.15)	5.45	<.001
Year 2020	1.96 (0.15)	12.97	<.001
Year 2021	1.97 (0.15)	13.20	<.001
Year 2022	2.40 (0.14)	17.73	<.001
Year 2023	0.17 (0.13)	1.37	.17
Year 2024	1.57 (0.13)	12.14	<.001
Year 2025	2.75 (0.14)	20.05	<.001
ρ (spatial lag)	0.12 (0.04)	3.13	.002

^a^ All covariates were obtained from County Health Rankings & Roadmaps releases (2015–2025), derived from the Behavioral Risk Factor Surveillance System, Centers for Medicare & Medicaid Services, Area Health Resources Files, National Center for Health Statistics, and American Community Survey, as documented by County Health Rankings & Roadmaps.

^b^ N = 899 county-year observations. Pseudo *R* ^2^ = 0.878. Spatial pseudo *R* ^2^ = 0.878.

^c^ Coefficients represent direct effects; higher values indicate worse health-related quality of life.

^d^ 2015 is reference category.

## References

[1] US Department of Health and Human Services, Centers for Disease Control and Prevention, National Center for Chronic Disease Prevention and Health Promotion, Division of Adult and Community Health. Measuring Healthy Days: Population Assessment of Health-Related Quality of Life. Centers for Disease Control and Prevention; 2000.

[2] Kindig DA , Cheng ER . Even as mortality fell in most US counties, female mortality nonetheless rose in 42.8 percent of counties from 1992 to 2006. *Health Aff (Millwood).* 2013;32(3):451–458. 10.1377/hlthaff.2011.0892 23459723

[3] Moriarty DG , Zack MM , Kobau R . The Centers for Disease Control and Prevention’s Healthy Days Measures — population tracking of perceived physical and mental health over time. *Health Qual Life Outcomes.* 2003;1(1):37. 10.1186/1477-7525-1-37 14498988 PMC201011

[4] County Health Rankings & Roadmaps. 2025 County Health Rankings National Findings Report. University of Wisconsin Population Health Institute. 2025. https://www.countyhealthrankings.org

[5] Mississippi State Department of Health. State of the State: Annual Mississippi Health Disparities and Inequities Report. Office of Health Surveillance & Research; 2023.

[6] Dwyer-Lindgren L , Bertozzi-Villa A , Stubbs RW , Morozoff C , Mackenbach JP , van Lenthe FJ , . Inequalities in life expectancy among US counties, 1980 to 2014: temporal trends and key drivers. *JAMA Intern Med.* 2017;177(7):1003–1011. 10.1001/jamainternmed.2017.0918 28492829 PMC5543324

[7] Zullig KJ , Hendryx M . Health-related quality of life among central Appalachian residents in mountaintop mining counties. *Am J Public Health.* 2011;101(5):848–853. 10.2105/AJPH.2010.300073 21421943 PMC3076406

[8] University of Wisconsin Population Health Institute. *Building Power for Health and Equity: 2025 County Health Rankings & Roadmaps Report.* Accessed May 13, 2026. https://www.countyhealthrankings.org/sites/default/files/media/document/2025%20CHRR%20Report_0.pdf

[9] Pierannunzi C , Xu F , Wallace RC , Garvin W , Greenlund KJ , Bartoli W , . A methodological approach to small area estimation for the Behavioral Risk Factor Surveillance System. *Prev Chronic Dis.* 2016;13:E91. 10.5888/pcd13.150480 27418213 PMC4951081

[10] Centers for Disease Control and Prevention. Behavioral Risk Factor Surveillance System: Overview. 2024. https://www.cdc.gov/brfss/annual_data/2024/pdf/Overview_2024-508.pdf

[11] Lee JE , Sung J , Lee J-Y , Baner Y , Offiah E , Hays A . Spatiotemporal modeling of premature mortality in Mississippi: a county-level analysis of years of potential life lost, 2015–2025. *Prev Chronic Dis.* 2026;23. 10.5888/pcd23.260035 PMC13475258 42594183

[12] Anselin L . Local indicators of spatial association — LISA. *Geogr Anal.* 1995;27(2):93–115. 10.1111/j.1538-4632.1995.tb00338.x

[13] Moran PAP . Notes on continuous stochastic phenomena. *Biometrika.* 1950;37(1–2):17–23. 10.1093/biomet/37.1-2.17 15420245

[14] Getis A , Ord JK . The analysis of spatial association by use of distance statistics. *Geogr Anal.* 1992;24(3):189–206. 10.1111/j.1538-4632.1992.tb00261.x

[15] Esri. Space-Time Pattern Mining: Hot Spot Analysis. Environmental Systems Research Institute; 2025.

[16] Lee JE , Sung JH , Lee J-Y . Bayesian hierarchical spatiotemporal models outperform spatial autoregressive models in predictive stability: analysis of Mississippi county-level premature mortality, 2015–2025 County Health Rankings releases. Published online January 26, 2026. 10.20944/preprints202601.1958.v1

[17] Egede LE , Walker RJ , Williams JS . Addressing structural inequalities, structural racism, and social determinants of health: a vision for the future. *J Gen Intern Med.* 2024;39(3):487–491. 10.1007/s11606-023-08426-7 37740168 PMC10897090

[18] Lawrence WR , Hong HG , Williams F , Dyer Z , Bergeron NQ , Brewer LC , . Manifestations of structural racism and inequities in cardiovascular health across US neighborhoods. *JAMA Health Forum.* 2025;6(10):e253864. 10.1001/jamahealthforum.2025.3864 41171261 PMC12579350

[19] Williams DR , Lawrence JA , Davis BA . Racism and health: evidence and needed research. *Annu Rev Public Health.* 2019;40(1):105–125. 10.1146/annurev-publhealth-040218-043750 30601726 PMC6532402

[20] Coronavirus Aid, Relief, and Economic Security (CARES) Act, HR 748, 116th Cong (2020). Public Law No. 116-136. Accessed March 20, 2026. https://www.congress.gov/116/bills/hr748/BILLS-116hr748enr.pdf

[21] US Department of the Treasury. State and local fiscal recovery funds. 2021. Accessed May 13, 2026. https://home.treasury.gov/policy-issues/coronavirus/assistance-for-state-local-and-tribal-governments/state-and-local-fiscal-recovery-funds

[22] Lee JGL , Henriksen L , Rose SW , Moreland-Russell S , Ribisl KM . A systematic review of neighborhood disparities in point-of-sale tobacco marketing. *Am J Public Health.* 2015;105(9):e8–e18. 10.2105/AJPH.2015.302777 26180986 PMC4529779

[23] Centers for Disease Control and Prevention, Office on Smoking and Health. Summary of Scientific Evidence: Comprehensive Tobacco Control Programs, June 2021. US Department of Health and Human Services, Centers for Disease Control and Prevention; 2021. Accessed March 10, 2026. https://www.cdc.gov/tobacco/media/pdfs/2024/07/comprehensive-TCP-508.pdf

[24] American Nonsmokers’ Rights Foundation. (2024). Lists & maps. U.S. Tobacco Control Laws Database. Accessed March 14, 2026. https://no-smoke.org/materials-services/lists-maps

[25] Reich BJ , Hodges JS , Zadnik V . Effects of residual smoothing on the posterior of the fixed effects in disease-mapping models. *Biometrics.* 2006;62(4):1197–1206. 10.1111/j.1541-0420.2006.00617.x 17156295

[26] Paciorek CJ . The importance of scale for spatial-confounding bias and precision of spatial regression estimators. *Stat Sci.* 2010;25(1):107–125. 10.1214/10-STS326 21528104 PMC3082155

[27] Sommers BD , Maylone B , Blendon RJ , Orav EJ , Epstein AM . Three-year impacts of the Affordable Care Act: improved medical care and health among low-income adults. *Health Aff (Millwood).* 2017;36(6):1119–1128. 10.1377/hlthaff.2017.0293 28515140

[28] Cohen J . Statistical Power Analysis for the Behavioral Sciences. 2nd ed. Lawrence Erlbaum Associates; 1988.

[29] Zhang X , Holt JB , Yun S , Lu H , Greenlund KJ , Croft JB . Validation of multilevel regression and poststratification methodology for small area estimation of health indicators from the Behavioral Risk Factor Surveillance System. *Prev Chronic Dis.* 2015;182(2):127–137. 10.1093/aje/kwv002 25957312 PMC4554328

[30] Openshaw S . The Modifiable Areal Unit Problem. GeoBooks; 1984.

[31] Tourangeau R , Rips LJ , Rasinski K . The Psychology of Survey Response. Cambridge University Press; 2000. 10.1017/CBO9780511819322

[32] Pierce M , McManus S , Hope H , Hotopf M , Ford T , Hatch SL , . Mental health responses to the COVID-19 pandemic: a latent class trajectory analysis using longitudinal UK data. *Lancet Psychiatry.* 2021;8(7):610–619. 10.1016/S2215-0366(21)00151-6 33965057 PMC9764381

[33] MacCallum RC , Zhang S , Preacher KJ , Rucker DD . On the practice of dichotomization of quantitative variables. *Psychol Methods.* 2002;7(1):19–40. 10.1037/1082-989X.7.1.19 11928888

[34] Bell A , Jones K . Explaining fixed effects: random effects modeling of time-series cross-sectional and panel data. *Political Sci Res Methods.* 2015;3(1):133–153. 10.1017/psrm.2014.7

[35] Subramanian SV , Jones K , Duncan C . Multilevel methods for public health research. In: Kawachi I , Berkman LF , eds. Neighborhoods and Health. Oxford University Press; 2003:65–111. 10.1093/acprof:oso/9780195138382.003.0004

